# Draft Genome Sequences of Two ATCC *Staphylococcus aureus* subsp. *aureus* Strains

**DOI:** 10.1128/genomeA.00618-17

**Published:** 2017-07-06

**Authors:** Catherine Putonti, Laurynas Kalesinskas, Evan Cudone, Kathleen C. Engelbrecht, David W. Koenig, Alan J. Wolfe

**Affiliations:** a Bioinformatics Program, Loyola University Chicago, Chicago, Illinois, USA; b Department of Biology, Loyola University Chicago, Chicago, Illinois, USA; c Department of Computer Science, Loyola University Chicago, Chicago, Illinois, USA; d Department of Microbiology and Immunology, Stritch School of Medicine, Health Sciences Division, Loyola University Chicago, Maywood, Illinois, USA; e Corporate Research and Engineering, Kimberly-Clark Corporation, Neenah, Wisconsin, USA

## Abstract

Draft genome sequences for *Staphylococcus aureus* subsp. *aureus* Rosenbach ATCC 14458 and ATCC 27217 strains were investigated. The genome sizes were 2,880,761 bp and 2,759,100 bp, respectively. Strain ATCC 14458 was assembled into 39 contigs, including 3 plasmids, and strain ATCC 27217 was assembled into 25 contigs, including 2 plasmids.

## GENOME ANNOUNCEMENT

As part of an attempt to generate complete genome sequences for a subset of strains in the ATCC collection, we report here the genome sequences and annotations of *Staphylococcus aureus* subsp. *aureus* Rosenbach ATCC 14458, isolated from the feces of a child with acute nonspecific diarrhea (https://www.atcc.org/Products/All/14458.aspx), and *S. aureus* subsp. *aureus* Rosenbach ATCC 27217, isolated from a nurse’s nares (https://www.atcc.org/Products/All/27217.aspx). 

The purchased culture isolates were grown on 5% sheep blood agar (BD BBL prepared plated medium) under 5% CO_2_ at 35°C for 48 h. The following procedure was used for genomic DNA extraction of each strain. Cells were resuspended in 0.5 ml of DNA extraction buffer (20 mM Tris-HCl, 2 mM EDTA, 1.2% Triton X-100 [pH 8]), followed by the addition of 50 µl of lysozyme (20 mg/ml), 30 µl of mutanolysin, and 5 µl of RNase (10 mg/ml), and then incubated at 37°C for 1 h. Next, 80 µl of 10% SDS and 20 µl of proteinase K were added and incubated for 2 h at 55°C. Two hundred ten microliters of 6 M NaCl and 700 µl of phenol-chloroform were added and incubated with rotation for 30 min. The solutions were centrifuged at 13,500 rpm for 10 min, and the aqueous phase was extracted. An equivalent volume of isopropanol was added, followed by incubation for 10 min and centrifugation at 13,500 rpm for 10 min. The supernatant was decanted and the DNA pellet precipitated using 600 µl of 70% ethanol. Following ethanol evaporation, the DNA pellet was resuspended in Tris-EDTA (TE) and stored at −20°C.

The genomic DNA isolated from each strain was diluted in water to a concentration of 0.2 ng/µl, as measured by a fluorometric-based method (Life Technologies, Inc.). Library preparation of 1 ng of input DNA was performed using the Nextera XT DNA library preparation kit. Sequencing was conducted on the Illumina MiSeq platform using the MiSeq reagent kit version 2 (500 cycles). For each strain, reads were filtered using BBDuk (http://sourceforge.net/projects/bbmap/), assembled using SPAdes (version 3.5) (1), scaffolded using SSPACE (2), and annotated using Peasant (3). Clustered regularly interspaced short palindromic repeat (CRISPR) arrays were identified using CRISPRFinder (4).

Here, we present a summary of the two draft genome sequences produced. For the *S. aureus* subsp. *aureus* Rosenbach ATCC 14458 strain, 1,899,308 paired-end reads were generated and assembled into 39 contigs (*N*_50_, 818,972 bp; coverage, 292.8×), including three contigs (46,194, 4,556, and 4,442 bp in length) corresponding to individual plasmids. Six rRNA genes, 51 tRNA genes, 2,753 protein-coding sequences, and eight CRISPR arrays were identified. For the ATCC 27217 strain, 1,579,161 paired-end reads were produced and assembled into 25 contigs (*N*_50_, 765,849 bp; coverage, 248.2×), including two contigs (23,114 and 4,625 bp in length) from plasmids. Nine rRNA genes, 57 tRNA genes, 2,564 protein-coding sequences, and 10 CRISPR arrays were detected. The draft genome sequences of the ATCC 14458 and ATCC 27217 strains total 2,880,761 bp (G+C content, 32.71%) and 2,759,100 bp (G+C content, 37.73%), respectively.

### Accession number(s).

The draft whole-genome project for *Staphylococcus aureus* strains ATCC 14458 and ATCC 27217 has been deposited at DDBJ/EMBL/GenBank under accession numbers NARA00000000 and NARB00000000, respectively. Raw sequence reads are deposited at DDBJ/EMBL/GenBank under accession numbers SRR5364301 and SRR5364258, respectively.
